# Implications of IFNγ SNP rs2069705 in primary Sjögren’s syndrome: transcriptional activation and B cell infiltration

**DOI:** 10.1152/ajpcell.00661.2023

**Published:** 2024-02-26

**Authors:** Xi Chen, Min Li, Honglin Li, Miao Liu, Jianrong Su, Yuzhu Ji

**Affiliations:** ^1^Department of Clinical Laboratory, Mianyang Central Hospital, Mianyang, China; ^2^Department of Immunology, Mianyang Central Hospital, Mianyang, China; ^3^Department of Pathology, Mianyang Central Hospital, Mianyang, China

**Keywords:** IFNγ, primary Sjögren’s syndrome, SNP site, susceptibility

## Abstract

Primary Sjögren’s syndrome (pSS) is characterized by its autoimmune nature. This study investigates the role of the IFNγ SNP rs2069705 in modulating the susceptibility to pSS. Differential expression of IFNγ and BAFF was analyzed using the GEO database’s mRNA microarray GSE84844. Genotyping of the IFNγ SNP rs2069705 was conducted via the dbSNP website. The JASPAR tool was used for predicting transcription factor bindings. Techniques such as dual-luciferase reporter assays, Chromatin immunoprecipitation, and analysis of a pSS mouse model were applied to study gene and protein interactions. A notable increase in the mutation frequency of IFNγ SNP rs2069705 was observed in MNCs from the exocrine glands of pSS mouse models. Bioinformatics analysis revealed elevated levels of IFNγ and BAFF in pSS samples. The model exhibited an increase in both CD20+ B cells and cells expressing IFNγ and BAFF. Knocking down IFNγ resulted in lowered BAFF expression and less lymphocyte infiltration, with BAFF overexpression reversing this suppression. Activation of the Janus kinase (JAK)/STAT1 pathway was found to enhance transcription in the BAFF promoter region, highlighting IFNγ’s involvement in pSS. In addition, rs2069705 was shown to boost IFNγ transcription by promoting interaction between its promoter and STAT4. SNP rs2069705 in the IFNγ gene emerges as a pivotal element in pSS susceptibility, primarily by augmenting IFNγ transcription, activating the JAK/STAT1 pathway, and leading to B-lymphocyte infiltration in the exocrine glands.

**NEW & NOTEWORTHY** The research employed a combination of bioinformatics analysis, genotyping, and experimental models, providing a multifaceted approach to understanding the complex interactions in pSS. We have uncovered that the rs2069705 SNP significantly affects the transcription of IFNγ, leading to altered immune responses and B-lymphocyte activity in pSS.

## Graphical abstract

**Figure:**
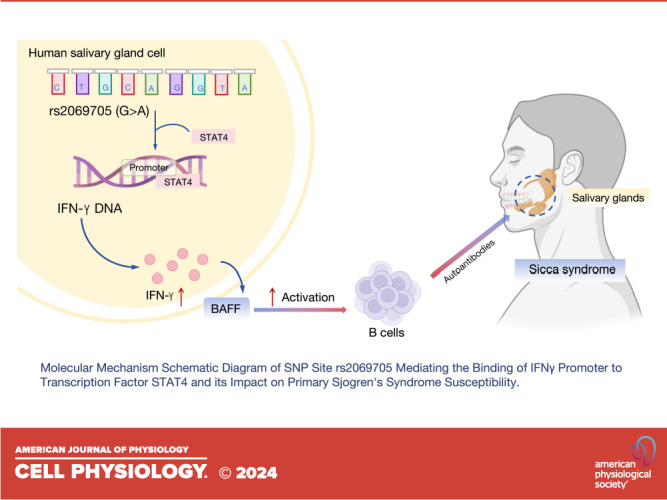

## INTRODUCTION

Primary Sjögren’s syndrome (pSS) is an autoimmune disorder characterized by lymphocytic infiltration and dysfunction of the salivary and lacrimal glands (1). This condition is primarily identified by the presence of dryness in the mouth and eyes, often accompanied by other immune system abnormalities (2). Despite extensive research, the exact cause of pSS remains incompletely understood, with multiple factors, including immune dysregulation, environmental influences, and genetic predispositions, contributing to its development (3, 4). Patients with pSS exhibit various irregularities in their immune system, including dysfunction of B and T cells and atypical immunoglobulin profiles (5). In addition, several cytokines and signaling pathways, such as IFN, NF-κB, Toll-like receptors, and others, have been implicated in the pathogenesis of pSS (6–8). Furthermore, specific genetic variants in genes like PTPN22, PADI4, and STAT4 have been shown to influence susceptibility and progression of pSS (9–11).

The pathogenesis of pSS is multifaceted, necessitating a comprehensive investigation of its underlying mechanisms. Although the exact cause of pSS is multifactorial, genetics plays a significant role, with various genetic factors contributing to susceptibility and progression (12). Recent advances in genomics have highlighted the importance of single nucleotide polymorphisms (SNPs) in understanding the genetic basis of autoimmune diseases, including pSS (13). Notably, recent genomic association studies have identified the rs2069705 locus as a robust susceptibility marker for pSS (9). This genetic variant is located within the promoter region of IFNγ, a key player in pSS pathogenesis (14). Research suggests that variations at the rs2069705 locus influence transcriptional activity and IFNγ expression levels within the IFNγ promoter region (15). In addition, the transcription factor STAT4 plays a central role in the IFNγ signaling pathway, crucial for immune regulation and inflammatory responses (10). STAT4 is also prominently implicated in the pathogenesis of pSS (6, 10). Elevated STAT4 mRNA expression levels have been observed in peripheral blood mononuclear cells of patients with pSS, correlating with disease severity (16). Notably, variants at the rs2069705 locus may influence the binding affinity of STAT4 to the IFNγ promoter region, thereby modulating IFNγ expression levels and susceptibility to pSS (17).

Therefore, it is evident that the rs2069705 locus regulates the interaction between the IFNγ promoter and the transcription factor STAT4, ultimately affecting IFNγ expression levels. This molecular mechanism is pivotal in the context of pSS susceptibility. A comprehensive functional investigation of the SNP locus rs2069705 within IFNγ is anticipated to advance our understanding of pSS pathogenesis, offering novel molecular markers and therapeutic targets for the early diagnosis and treatment of this condition.

## MATERIALS AND METHODS

### Bioinformatics Analysis to Screen for Differentially Expressed Genes in pSS Samples

The mRNA expression chip GSE84844 related to pSS was downloaded from the Gene Expression Omnibus (GEO) database (https://www.ncbi.nlm.nih.gov/gds), which included 30 samples from patients with pSS and 30 samples from normal healthy individuals. Differential gene expression (DEG) was identified using the “limma” package in R language, with a threshold of *P* value < 0.01 for DEG selection. The base sequence upstream of 2KB from the IFNγ promoter was queried using the UCSC Genome browser (http://genome.ucsc.edu/). The position and genotype of the IFNγ SNP locus rs2069705 were analyzed using the UCSC Genome browser. The JASPAR tool (http://jaspar.genereg.net/) was used to predict the transcription factors binding to the IFNγ SNP rs2069705. Consent for publication was obtained from the participants.

### Construction of a pSS Mouse Model

A total of 65 female C57BL/6N mice weighing 20–25 g, at 6 wk of age, were obtained from Beijing VITO Laboratory Animal Technology Co., Ltd (China) (Cat. No.: 213) and housed in an SPF-grade animal facility for breeding. The study was conducted in accordance with relevant guidelines and approved by our institutional Animal Ethics Committee.

Twenty mice were randomly selected as controls, while the remaining mice were used to establish a pSS mouse model through immunization induced by submandibular gland (SMG) autoantigens. The SMG autoantigen was prepared on ice: five C57BL/6N mice were euthanized by cervical dislocation, and the SMGs were immediately aseptically removed, dissected free of fat and connective tissue, weighed, minced, and then homogenized with 0.1 mol/L pH 7.2 phosphate buffer. The supernatant was separated after centrifugation and dissolved in PBS to adjust the concentration to 800 μg/mL. It was then emulsified in equal amounts of complete Freund’s adjuvant to a final concentration of 400 μg/mL. The pSS model group mice (40 in total) received a subcutaneous injection of 0.1-mL autoantigen emulsified with complete Freund’s adjuvant on *day 1*, followed by a booster injection with the same dosage on *day 14*. The control group mice (20 in total) received subcutaneous injections of 0.1-mL PBS plus adjuvant on *day 1* and *day 14* (after frequency analysis, 10 mice from each group were selected for other tissue experiments).

The remaining pSS mice were randomly divided into two groups: pSS + sh-IFNγ group, where the pSS mice received submandibular gland injections of 50-μL sh-IFNγ lentivirus (1 × 10^8^ TU/mL) every 3 days for a total of three times, and pSS + sh-IFNγ + rmBAFF group, where the pSS mice received submandibular gland injections of 50-μL sh-IFNγ lentivirus (1 × 10^8^ TU/mL) and 3 μg rmBAFF every 3 days for a total of three times. At the end of the sixth week, the mice were euthanized, and the exocrine gland tissues were collected for subsequent experiments.

### H&E Staining

Exocrine gland tissues from the pSS mouse model and its control group were fixed in 4% paraformaldehyde for 36 h, embedded in paraffin, and sectioned. After routine deparaffinization to water, the sections were stained with hematoxylin for 4 min, rinsed, differentiated in hydrochloric alcohol for 10 s, rinsed, blued in ammonia water for 10 min, stained with eosin solution for 2 min, dehydrated in graded alcohol for 1 min each, cleared in xylene twice for 1 min each, and observed using an optical microscope (Model DMM-300D, Shanghai Caikang Optical Instrument Co., Ltd, Shanghai, China). For quantification, the H&E-stained slides were scanned using an Aperio CS2 digital pathology scanner and analyzed with Aperio ImageScope software (Leica Biosystems, Buffalo Grove, IL) to measure the number of lymphocytic foci infiltrating exocrine gland tissues under low magnification and the number of infiltrating lymphocytes in each lymphocytic focus under high magnification (18).

### Immunohistochemical Staining

Exocrine gland tissue blocks from the pSS mouse model and its control group were fixed in 4% paraformaldehyde for 12 h, underwent routine deparaffinization with xylene, and were subjected to gradient alcohol hydration (anhydrous ethanol, 95% ethanol, 75% ethanol for 3 min each). The tissues were then boiled in 0.01 M citrate buffer for 15–20 min, cooled to room temperature, and washed with PBS. Goat serum blocking solution was added, and after 20 min at room temperature, excess liquid was removed. Primary antibodies CD20 (1:100, ab64088, Abcam, UK), IFNγ (1:200, 15365-1-AP, ProteinTech), STAT4 (1:100, ab284412, Abcam, UK), and BAFF (ab5965, 1:2,000, Abcam, UK) were separately added and incubated at room temperature for 1 h, followed by PBS washing. Biotin-labeled goat anti-rabbit IgG (ab6728, 1:1,000, Abcam, UK) was then added, and the slides were incubated in a humid chamber at room temperature for 30 min. After PBS washing, SP (streptavidin-peroxidase) was added, and the slides were left at 37°C for 30 min. Following PBS washing, DAB (Cat. No.: DA1015, Beijing Sulaibao Biotechnology Co., Ltd, Beijing, China) was applied for color development for 5–10 min, followed by a 10-min rinse with running water to stop the reaction. Subsequently, the sections were counterstained with hematoxylin for 2 min, subjected to hydrochloric acid alcohol differentiation, rinsed with running water for 10 min, dehydrated, cleared, and coverslipped for microscopic observation. Cytoplasmic staining with brown granules was considered positive. Ten random fields at ×200 magnification were selected from the sections, with 100 cells counted in each field to calculate the percentage of positively stained cells, and the mean value was determined (19).

### PCR-HRM Technique for SNP Genotyping

Individual nuclear cell DNA was extracted from exocrine gland tissues of pSS mice. Primers for amplifying the IFNγ SNP rs2069705 site (F: 5′- AATTCCTAGCACTTTATGAGG-3′, R: 5′- AGTCTTGCTCTGTCACCCA-3′) were designed using primer design software and synthesized by Shanghai Sangon Biotech Co., Ltd (Shanghai, China).

#### PCR reactions and cycling conditions.

Predenaturation at 95°C for 10 min, denaturation at 92°C, annealing at 57°C, and extension at 72°C for 30 s each, for a total of 35 cycles. After PCR, the reaction products were transferred to a 96-well plate, denatured at 95°C for 30 s, followed by reannealing at 25°C, and then placed in a LightScanner instrument. The scanning temperature range was set from 72°C to 95°C, and the program was run to collect melting curves. The LightScanner software was used to analyze the melting curves for SNP genotyping (20).

### Isolation and Stimulation of MNCs and B Lymphocytes

Exocrine gland tissues from normal control mice and pSS mice were minced into small pieces, homogenized, and subjected to ultrasonic disruption. Single-cell suspensions were obtained through enzymatic digestion, followed by lymphocyte separation. Mononuclear cells (MNCs) were isolated, and lymphocyte counting was performed. Cell concentration was adjusted to 5 × 10^6^ cells/mL under a microscope, and CD20 antibody (1:40, ab64088, Abcam, UK) was added. CD20+ B-lymphocytes were then isolated using magnetic bead separation.

MNC cells isolated from the lip gland were divided into groups for treatment: blank group, 5 ng/mL IFNγ group, 10 ng/mL IFNγ group, and 100 ng/mL IFNγ group. The cells were continuously stimulated for 2 days, and cell culture supernatants were collected. Quantitative expression of IFNγ and soluble BAFF cytokines in the cell culture supernatant was measured using human IFNγ assay kit (JL22046, Shanghai Jianglai Industrial Co., Ltd, Shanghai, China) and human BAFF assay kit (JL19053, Shanghai Jianglai Industrial Co., Ltd, Shanghai, China).

### MNC Cell Culture and Grouping

MNC cells were cultured using specialized culture media (Cat. Nos.: 165863 and MCM-0233, Zhejiang, China, Ningbo Mingzhou Biological Technology Co., Ltd). MNC cells were divided into groups: *1*) pretreatment of MNC cells with 1.253 μM JAK/STAT1 inhibitor PF-04965842 for 1 h, followed by stimulation with 100 ng/mL IFNγ for 48 h. Western blotting was performed to detect phosphorylated JAK and STAT1 proteins, and ELISA was used to measure BAFF expression in the cell culture supernatant. *2*) Overexpression of STAT4 (or T-Bet) in MNC cells, dividing the cells into oe-NC and oe-STAT4 groups. Plasmid pCMV6-AC-GFP was purchased from Fenghui Biotechnology (Cat. No.: FH1215, Changsha, Hunan), and transfection was performed using the Lipofectamine 2,000 kit (Cat. No.: 11668019, Thermo Fisher). Plasmids and Lipofectamine 2,000 were diluted in 250 μL serum-free Opti-MEM medium (Cat. No.: 31985062, Thermo Fisher), and then mixed and incubated at room temperature for 20 min. The mixture was added to the culture wells and placed in a 37°C, 5% CO_2_ incubator. After 6 h, the medium was replaced with complete culture medium, and the cells were further cultured for 48 h. Transfection efficiency was checked, and the cells were used for subsequent experiments.

### Western Blot

Total protein was extracted using RIPA lysis buffer containing PMSF (P0013C, Beyotime, Shanghai, China). SDS-PAGE gel electrophoresis was performed, and the proteins were transferred to a PVDF membrane. The membrane was blocked with 5% skim milk at room temperature for 1 h. Then, the PVDF membrane was incubated with diluted primary antibodies: p-JAK1 (1:1,000, ab138005, Abcam, Cambridge, UK), STAT1 (1:1,000, ab239360, Abcam, Cambridge, UK), p-STAT1 (1:2,000, ab109461, Abcam, Cambridge, UK), and BAFF (1:2,000, ab8396, Abcam, Cambridge, UK), with GAPDH (ab9485, 1:2,500, Abcam, Cambridge, UK) as an internal reference. The membrane was then incubated with HRP-labeled secondary antibody goat anti-rabbit IgG H&L (HRP) (ab97051, 1:2,000, Abcam, Cambridge, UK) for 1 h. The ECL fluorescence detection kit [Cat. No. abs920, Aibixing (Shanghai) Testing & Photographic Co., Ltd] was used, and the relative protein content was analyzed using Quantity One v4.6.2 software, represented by the grayscale value of the corresponding protein band/GAPDH protein band (21). The experiment was repeated three times, and the average value was taken.

### RT-qPCR

Total RNA from cells was extracted using TRIzol (Cat. No.: 15596026, Thermo Fisher). For mRNA detection, the PrimeScript RT reagent kit (Takara Code: RR047A, Takara, Japan) was used for reverse transcription to obtain cDNA. SYBR Green (Perfect Real Time) kit (Takara, Japan) was used for sample loading. The 7500 Fast Real-time PCR System (Cat. No.: 4351106, Thermo Fisher) was used for RT-qPCR to detect mRNA expression levels. The primer sequences can be found in Table 1. The Ct value was measured in the exponential amplification stage, and the relative transcription level of the target gene was calculated using the relative quantitative method (2^−ΔΔCT^ method): ΔΔCt = ΔCt experimental group—ΔCt control group, ΔCt = Ct (target gene)— Ct (reference gene), and the relative transcription level of the target gene = 2^−ΔΔCt^.

**Table 1.** Primer sequences for RT-qPCR

Genes	Primer Sequences
GAPDH (mice)	F: 5′-CCCTTAAGAGGGATGCTGCC-3′
	R: 5′-TACGGCCAAATCCGTTCACA-3′
IFNγ (mice)	F: 5′-AGGAAGCGGAAAAGGAGTCG-3′
	R: 5′-GGAAGCACCAGGTGTCAAGT-3′
β-Actin (mice)	F: 5′-CATTGCTGACAGGATGCAGAAGG-3′
	R: 5′-TGCTGGAAGGTGGACAGTGAGG-3′
BAFF (mice)	F: 5′-TGAAACACCAACTATACAAAAAG-3′
	R: 5′-TCAATTCATCCCCAAAGACAT-3′
STAT4 (mice)	F: 5′-TCAGTGAGAGCCATCTTGGAGG-3′
	R: 5′-TGTAGTCTCGCAGGATGTCAGC-3′

BAFF, B-cell-activating factor; F, forward; GAPDH, glyceraldehyde-3-phosphate dehydrogenase; IFNγ, interferon-gamma; R, reverse; RT-qPCR, reverse transcription-quantitative polymerase chain reaction; STAT4, signal transducers and activators of transcription 4.

### Dual-Luciferase Reporter Gene Assay

The fluorescence enzyme reporter gene experiment validated the targeting relationships between STAT4 and IFNγ, as well as STAT1 and BAFF. First, fragment sequences were obtained for the binding of STAT4 with IFNγ SNP site ( TGAGTTAACTTC) and the GAS site ( GGAAAACCAAACAT) binding of STAT1 with BAFF. These sequences were cloned, and the SNP site A base was mutated to G ( TGAGTTAGCTTC) and the GAS site mutation sequence ( CATTTTAATTTGTA) was cloned as well. These sequences were inserted into the pGL3-Luciferase Reporter Vector (Promega, WI) vector, named pGL3-IFNγ-SNP-WT, pGL3-IFNγ-SNP-MT, GAS-WT, and GAS-MT, respectively.

#### Validation of STAT4 targeting binding to IFNγ SNP site.

oe-NC and oe-STAT4 were separately cotransfected with the luciferase reporter plasmids (pGL3-IFNγ-SNP-WT and pGL3-IFNγ-SNP-MT) into cells. The cells were lysed in 1× Passive lysis buffer, and Dual-Luciferase Reporter Assay System (Promega, USA) was used to measure the firefly luciferase enzyme activity, with Renilla luciferase activity as an internal reference.

#### Validation of STAT1 targeting binding to BAFF GAS site.

After treating the cells with JAK/STAT1 inhibitor PF-04965842 for 2 h, 100 ng/mL IFNγ and luciferase reporter plasmids (GAS-WT, GAS-MT) were added. After incubation for 48 h, the cells were lysed in 1× Passive lysis buffer, and Dual-Luciferase Reporter Assay System (Promega, USA) was used to measure the firefly luciferase enzyme activity, with Renilla luciferase activity as an internal reference (22).

### Chromatin Immunoprecipitation

The enrichment of STAT1 in the BAFF promoter region was detected using a chromatin immunoprecipitation (ChIP) kit (Cat. No.: KT101-02, SAB Biotherapeutics, Guangzhou, China). The steps were as follows: when the fusion degree of MNC cells reached 70%–80%, 1% formaldehyde was added at room temperature for 10 min to cross-link DNA and proteins in the cells. After cross-linking, the cells were randomly broken by ultrasonication, with each cycle consisting of 10 s of sonication and 10 s of interval, repeated 15 times, to break them into appropriate-sized fragments. After centrifugation at 13,000 rpm at 4°C, the supernatant was collected and divided into three tubes. The tubes were incubated at 4°C overnight with positive control antibody RNA polymerase II rabbit antibody (1:100, ab238146, Abcam, UK), negative control antibody rabbit anti-IgG (ab172730, 1:100, Abcam, UK), and target protein-specific antibody rabbit anti-STAT1 (1:100, ab239360, Abcam, UK). Protein Agarose/Sepharose was used to precipitate endogenous DNA-protein complexes. After brief centrifugation, the supernatant was discarded, and nonspecific complexes were washed. Cross-linking was reversed overnight at 65°C, and phenol/chloroform extraction was performed to purify and recover DNA fragments for qPCR to detect BAFF gene promoter fragments (23, 24).

### Statistical Analysis

Statistical analysis was performed using SPSS 21.0 (IBM, USA) statistical software. Measurement data were expressed as means ± standard deviation. Nonpaired *t* tests were used for intergroup comparisons, and one-way analysis of variance or repeated measures analysis of variance was used for comparisons between multiple groups. Pearson correlation analysis and Spearman rank correlation analysis were used to observe the correlation of indicators (25). *P* < 0.05 indicates statistical significance.

## RESULTS

### Significantly Increased Frequency of Mutations in the SNP Locus rs2069705 of IFNγ in MNC of Exocrine Gland Tissue from pSS Mice

Several studies have found an association between the rs2069705 single nucleotide polymorphism (SNP) in the IFNγ promoter and susceptibility to various autoimmune disorders (26, 27). However, there is no reported role of rs2069705 in the occurrence and development of primary Sjögren’s syndrome (pSS). Using Ficoll density gradient separation, we obtained mononuclear cells (MNC) from the exocrine gland tissues of 20 pSS mice and 20 normal control mice, extracted DNA, and detected the IFNγ SNP (rs2069705). As shown in Table 2, in the pSS group, the G allele frequency of the IFNγ rs2069705 SNP was significantly higher than that in the Control group (*P* = 0.0091).

**Table 2.** Genotyping of pSS group and Control group

Group	rs2069705		
	G/G	G/A	A/A
Control, *n* = 20	2	4	14
pSS, *n* = 20	10	4	6

pSS, primary Sjögren’s syndrome.

### High Expression of IFNγ and BAFF in Lacrimal Gland Tissue of pSS Mice and Correlation between IFNγ and BAFF Expression and B-Lymphocyte Infiltration

**Figure 1.**
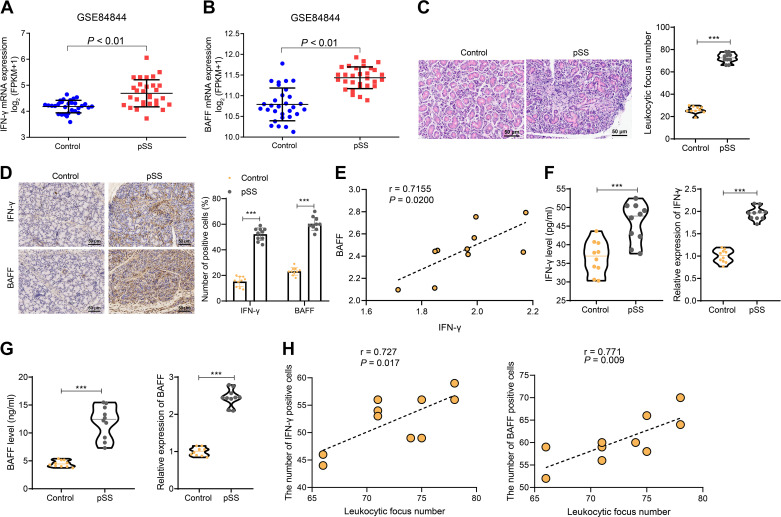
Expression of IFNγ and BAFF in pSS samples and their correlation with B-lymphocyte infiltration. *A*: differential analysis of IFN-γ mRNA expression in normal (*n* = 30) and pSS (*n* = 30) samples by microarray GSE84844. *B*: differential analysis of BAFF mRNA expression levels in normal (*n* = 30) and pSS (*n* = 30) samples by microarray GSE84844. *C*: the infiltration of lymphocytes in the exocrine gland tissues of normal control mice and pSS mice was observed using H&E staining at a magnification of 200 times (scale bar = 25 μm). *D*: immunohistochemical staining was performed to determine the positive expression rates of IFN-γ and BAFF proteins in the exocrine gland tissues of normal control mice and pSS mice at a magnification of 200 times (scale bar = 25 μm). *E*: the correlation between the expression of IFN-γ mRNA and BAFF mRNA was analyzed using the Pearson correlation coefficient method. *F*: the protein expression levels of IFN-γ in MNC cells isolated from the exocrine gland tissues of the Control group and pSS group were detected using ELISA and RT-qPCR. *G*: the protein expression levels of BAFF in MNC cells isolated from the exocrine gland tissues of the Control group and pSS group were detected using ELISA and RT-qPCR. *H*: Spearman hierarchical correlation analysis was performed to analyze the correlation between IFN-γ and the number of BAFF-positive cells and the number of B-lymphocyte infiltration foci; the quantitative data were represented using the means ± SD. The data between the two groups were compared using an independent *t* test. ***P < 0.001 compared between two groups; ns indicates *P* > 0.05 compared between two groups; all experiments were repeated three times; 10 mice per group mice. BAFF, B-cell-activating factor; ELISA, enzyme-linked immunosorbent assay; H&E, hematoxylin and eosin; IFNγ, interferon-gamma receptor; MNC, mononuclear cell; pSS, primary Sjögren’s syndrome; RT-qPCR, reverse transcription-quantitative polymerase chain reaction.

IFNγ is involved in the immune regulation of several autoimmune diseases, including pSS (28). B-cell-activating factor (BAFF) is key in promoting B-lymphocyte activation and survival in pSS, and IFNγ induces BAFF expression (29). We obtained the pSS-associated mRNA microarray GSE84844 from the GEO database, and the results of differential gene analysis showed (Fig. 1, *A* and *B*) that IFNγ and BAFF were highly expressed in pSS samples compared to normal samples.

In addition, we established a pSS mouse model. Hematoxylin and eosin (H&E) staining results displayed a significant infiltration of lymphocytes in the exocrine gland tissues of pSS mice compared to the control group (Fig. 1*C*). Immunohistochemistry results indicated a significant increase in the number of CD20^+^ B cells, as well as the presence of IFNγ and BAFF protein expression in the exocrine gland tissues of pSS mice compared to the control group (Fig. 1, *D* and *E*), showing a positive correlation.

Next, MNCs were magnetically sorted for CD20^+^ B-lymphocytes and quantified. Enzyme-linked immunosorbent assay (EISA) was used to detect the expression of IFNγ and BAFF in the cell culture supernatant of MNC. The results demonstrated elevated expression of both IFNγ and BAFF in the MNC cell culture supernatant of pSS mice compared to the control group (Fig. 1, *F* and *G*). Moreover, there was a positive correlation between the number of CD20^+^ B cells isolated from the exocrine gland tissues of mice and the expression of IFNγ and BAFF in the MNC cell culture supernatant (Fig. 1*H*).

### IFNγ Stimulates the Upregulation of BAFF Expression in MNC Cells of the Lacrimal Gland from the Exocrine Gland Tissues of pSS Mice

**Figure 2.**
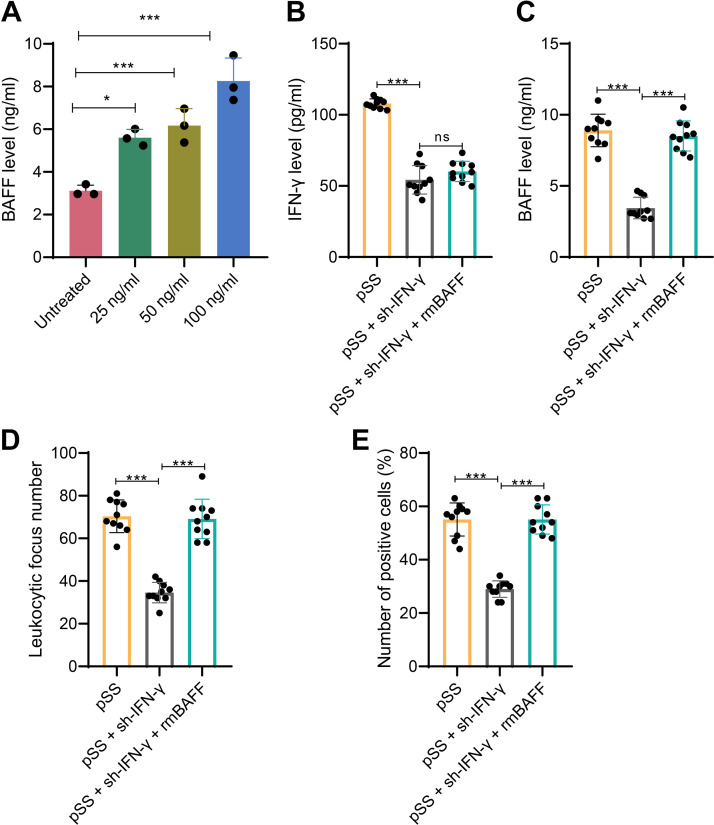
Effect of sh-IFNγ and rmBAFF on the expression of IFNγ and BAFF and B-lymphocyte infiltration in the exocrine gland tissue of pSS mice. *A*: different concentrations of IFN-γ were used to stimulate MNC cells derived from the exocrine gland tissues of pSS mice, and BAFF protein expression was measured using ELISA. *B*: the expression levels of IFN-γ in the exocrine gland tissues of pSS mice were detected by ELISA after knocking down IFN-γ or rmBAFF treatment. *C*: the expression levels of BAFF in the exocrine gland tissues of pSS mice were detected by ELISA after knocking down IFN-γ or rmBAFF treatment. *D*: lymphocyte infiltration in the exocrine gland tissues of each group of mice was examined using H&E staining. *E*: the number of positively stained cells expressing BAFF protein in the exocrine gland tissues of each group of mice was determined using immunohistochemical staining. The quantitative data were presented using the means ± SD. Multiple group data comparisons were conducted using one-way ANOVA analysis. *P < 0.05 compared to two groups; ***P < 0.001 compared to two groups; ns indicates *P* > 0.05 compared to two groups; 10 mice in each group. ANOVA, analysis of variance; BAFF, B-cell-activating factor; ELISA, enzyme-linked immunosorbent assay; H&E, hematoxylin and eosin; IFNγ, interferon-gamma receptor; MNC, mononuclear cell; pSS, primary Sjögren’s syndrome.

To further elucidate the role of IFNγ in the activation and survival of B lymphocytes in pSS, thereby resulting in immune system dysregulation including B cell dysfunction in patients with pSS, we stimulated MNC cells derived from salivary gland tissues of pSS mice with increasing concentrations of IFNγ. The cell culture supernatant was collected, and BAFF expression was detected using a BAFF ELISA assay. The results revealed a positive correlation between IFNγ concentration and BAFF protein expression in MNC cells (Fig. 2*A*), indicating that IFNγ can promote BAFF protein expression.

To investigate whether IFNγ promotes BAFF expression in vivo and if the expression of IFNγ and BAFF has an inducing effect on the disease progression in pSS mice, we performed IFNγ knockout in pSS mice and assessed the levels of BAFF expression and lymphocyte infiltration. The results indicated that compared to the pSS group, the positive rates of IFNγ (Fig. 2*B*) and BAFF (Fig. 2*C*) protein expression in the exocrine gland tissue of pSS + sh-IFNγ mice were significantly reduced, while the number of CD20^+^ B cells (Fig. 2*D*) in the exocrine gland tissue was significantly decreased. Moreover, supplementation of recombinant BAFF protein in IFNγ-knockout pSS mice resulted in a significant increase in the positive rate of BAFF protein expression in the exocrine gland tissue and a significant increase in the number of CD20^+^ B cells. Immunohistochemical semiquantitative analysis (Fig. 2*E*) demonstrated a decrease in lymphocyte infiltration levels in the exocrine gland tissue of IFNγ-knockout pSS mice and an increase in the number of lymphocytic infiltration foci and B cells upon supplementation of BAFF in these mice.

The above results showed that the knockdown of IFNγ significantly reduced BAFF expression and inhibited lymphocyte infiltration in the exocrine gland tissues of pSS mice, and overexpression of BAFF reversed the inhibitory effect of the knockdown of IFNγ.

### IFNγ Upregulates BAFF Expression through Activation of the JAK/STAT1 Pathway

**Figure 3.**
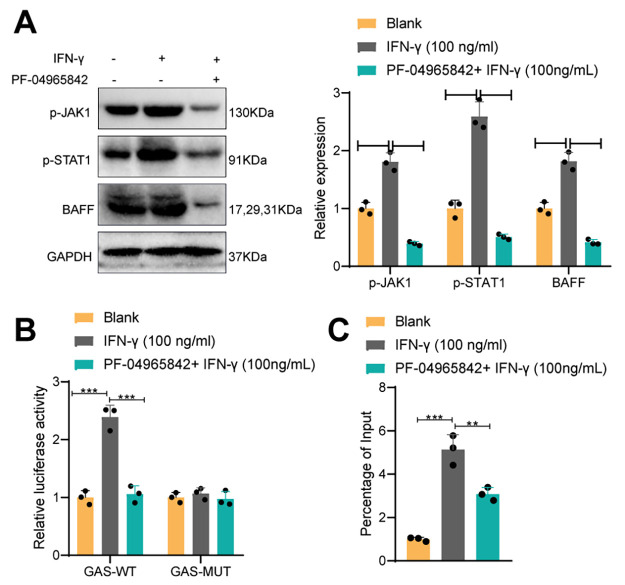
Effect of IFNγ regulation of JAK/STAT1 signaling pathway on BAFF expression in pSS. *A*: Western blot assay for the effect of IFNγ (100 ng/mL) and JAK-specific inhibitor (PF-04965842) on the expression of p-JAK1, p-STAT1, and BAFF in MNC cells. *B*: dual luciferase assay to verify the effect of IFNγ (100 ng/mL) and JAK-specific inhibitor (PF-04965842) on BAFF promoter activity. *C*: ChIP experiments on the effect of IFNγ (100 ng/mL) and JAK-specific inhibitor (PF-04965842) on STAT1 enrichment in the BAFF promoter region. The measurements were expressed as means ± SD, and multiple comparisons were performed using one-way analysis of variance (ANOVA). **P < 0.01 compared between the two groups; ***P < 0.001 compared between two groups; ns denotes *P* > 0.05 compared between two groups; all experiments were repeated three times. BAFF, B-cell-activating factor; ChIP, chromatin immunoprecipitation; IFNγ, interferon-gamma receptor; JAK, Janus kinase; MNC, mononuclear cell; pSS, primary Sjögren’s syndrome; STAT1, signal transducer and activator of transcription 1.

Previous studies have shown that IFNγ promotes the expression of BAFF in B cells by activating the JAK/STAT1 signaling pathway, and this regulatory effect is related to the pathogenesis of pSS (30). To further verify whether IFNγ in pSS promotes the expression of BAFF by regulating the JAK/STAT1 signaling pathway, we treated MNC cells from the exocrine glands of pSS mice with a JAK-specific inhibitor (PF-04965842) and measured the activation of the JAK/STAT1 signaling pathway biomarkers p-JAK1 and p-STAT1, as well as the expression levels of BAFF. Western blot analysis results (Fig. 3*A*) showed that the addition of IFNγ stimulation significantly increased the expression levels of p-JAK1, p-STAT1, and BAFF in MNC cells from the exocrine glands of pSS mice. However, pretreatment with the JAK inhibitor followed by IFNγ stimulation resulted in reduced expression levels of p-JAK1, p-STAT1, and BAFF compared to the previous group. These results indicate that the JAK inhibitor inhibits the activation of the JAK-STAT1 phosphorylation pathway induced by IFNγ and the expression of BAFF.

We further investigated how STAT1, activated by IFNγ, promotes the expression of BAFF. We found that STAT1 can bind to the GAS site in the BAFF promoter region and enhance its transcription. To validate the binding of activated STAT1 (induced by IFNγ) and inactivated STAT1 (induced by PF-04965842) to the BAFF GAS site, we performed dual-luciferase and CHIP experiments. First, we obtained the fragment sequences of the GAS site ( GGAAAACCAAACAT) where STAT1 binds to BAFF and cloned it along with the GAS site mutant sequence ( CATTTTAATTTGTA) in which the SNP site A is mutated to G. These sequences were inserted into the pGL3-Luciferase Reporter Vector (Promega, WI) and named GAS-WT and GAS-MT, respectively. The results of the dual-luciferase experiment (Fig. 3*B*) showed that in GAS-WT, the activity of the BAFF promoter significantly increased after IFN-γ treatment. Compared to the IFN-γ group, the activity of the BAFF promoter in the PF-04965842 + IFN-γ group significantly decreased. However, the activity of the BAFF promoter in GAS-MT was not affected. Subsequently, a ChIP experiment was performed using antibodies against STAT1. The results (Fig. 3*C*) demonstrated a significant increase in the enrichment of STAT1 in the BAFF promoter region after IFN-γ treatment. Compared to the IFN-γ group, the enrichment of STAT1 in the BAFF promoter region in the PF-04965842 + IFN-γ group significantly decreased.

Taken together, these results suggest that in pSS, IFNγ may induce the activation of STAT1 by activating the JAK/STAT1 signaling pathway, and activated STAT1 can bind to the GAS site in the BAFF promoter region, promoting its transcription and enhancing BAFF expression.

### IFNγ Could Promote IFNγ Transcription through SNP Locus rs2069705-Mediated Binding of the IFNγ Promoter to the Transcription Factor STAT4

**Figure 4.**
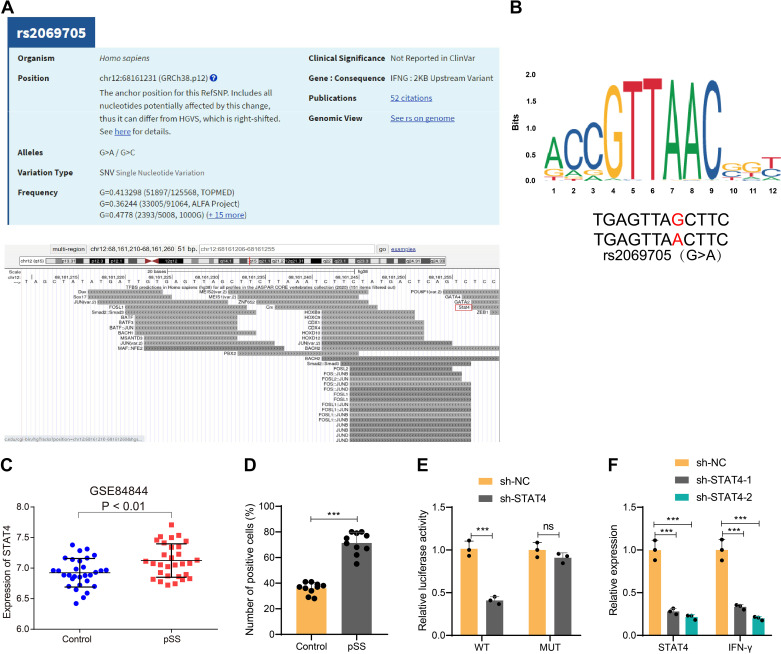
Validation of the regulatory relationship between the transcription factor STAT4 and IFNγ. *A*: the dbSNP website to analyze the genotype of rs2069705 and predict the transcription factors that can bind to the promoter region of IFNγ. *B*: JASPAR analysis tool to predict the upstream transcription factors of rs2069705 (G>A) sequence. *C*: differential analysis of STAT4 mRNA expression levels in normal (*n* = 30) and pSS (*n* = 30) samples by microarray GSE8484. *D*: immunohistochemical detection of STAT4 in pSS mice exocrine gland tissue (*n* = 10). *E*: dual luciferase assay to verify the regulation of transcription factor STAT4 and IFNγ. *F*: RT-qPCR to detect the effect of sh-STAT4 on the expression of IFNγ and STAT4 in MNC cells. The quantitative data were presented as means ± SD. Two-group comparisons were performed using an independent *t* test, while multiple group comparisons were analyzed using one-way analysis of variance (ANOVA). ***P < 0.001 compared between the two groups; ns indicates *P* > 0.05 compared between the two groups; cell experiments were repeated three times. IFNγ, interferon-gamma receptor; MNC, mononuclear cell; pSS, primary Sjögren’s syndrome; RT-qPCR, reverse transcription-quantitative polymerase chain reaction; STAT4, signal transducers and activators of transcription 4.

The experimental results from Figs. 1, 2, and 3 have confirmed that high expression of IFNγ is a significant inducer of susceptibility to primary Sjögren’s syndrome (pSS). In addition, the frequency of the SNP site rs2069705 in IFNγ is significantly increased in patients with pSS. To further investigate the role of the IFNγ promoter SNP site rs2069705 in the occurrence and development of pSS, we initially analyzed the genotype location of rs2069705 and predicted the transcription factors that can bind to the IFNγ promoter region using the UCSC Genome Browser website. We found that it includes the transcription factor STAT4 (Fig. 4*A*). STAT4 also plays a critical role in the onset of pSS (6, 10). Previous studies have shown that STAT4 mRNA expression levels are significantly elevated in peripheral blood mononuclear cells of patients with pSS and are associated with disease severity (16). Furthermore, the variation at the rs2069705 site can affect the binding affinity between STAT4 and the IFNγ promoter region, thereby influencing the expression levels of IFNγ and the susceptibility to pSS (17). Therefore, we focus on the transcription factor STAT4, and next, we use the JASPAR tool to predict the consensus sequence of the transcription factors that can bind to rs2069705 (Fig. 4*B*).

The differential analysis results of the GSE84844 chip demonstrate that STAT4 is highly expressed in pSS samples (Fig. 4*C*). Semiquantitative immunohistochemistry results also show high expression of STAT4 in salivary gland tissues of pSS mice (Fig. 4*D*). Thus, we hypothesize that the high expression of STAT4 in pSS, and the binding affinity of the transcription factor STAT4 to the IFNγ promoter region, is affected by the SNP site rs2069705 mutation, ultimately leading to the occurrence and development of pSS.

To validate this hypothesis, we conducted a dual-luciferase reporter assay in MNC cells to verify the targeted binding of the transcription factor STAT4 to the IFNγ SNP site. First, we constructed wild-type plasmids (p-GL3-IFNγ-SNP-WT) for IFNγ SNP (rs2069705), as well as mutant-type plasmids (p-GL3-IFNγ-SNP-MT) where the A base of the SNP site rs2069705 was mutated to G. The two reporter plasmids were cotransfected into MNC cells with silence plasmids sh-STAT4 and negative control plasmids using Lipofectamine. The results of the dual-luciferase reporter assay show (Fig. 4*E*) that in the cotransfection group with p-GL3-IFNγ-SNP-WT, compared to the sh-NC group, the luciferase activity significantly decreased in the sh-STAT4 group. However, in the cotransfection group with p-GL3-IFNγ-SNP-MT, there was no significant difference in luciferase activity. The RT-qPCR results show (Fig. 4*F*) that compared to the sh-NC group, the expression of IFNγ and STAT4 in MNC cells significantly decreased in the sh-STAT4 group.

These results indicate that the SNP site rs2069705 mediates the transcription of IFNγ by facilitating the binding between the IFNγ promoter region and the transcription factor STAT4.

## DISCUSSION

Despite extensive research efforts, the precise etiology of pSS remains enigmatic, with indications pointing toward the involvement of both environmental and genetic factors. Recent advancements in genomics have underscored the significance of SNPs in unraveling the genetic underpinnings of autoimmune diseases, including pSS (31). Genetic variations affecting the expression or function of IFNγ could have profound implications for immune regulation and the pathogenesis of autoimmune conditions like pSS. Investigating the role of the IFNγ SNP rs2069705 in pSS promises to provide pivotal insights into the intricate molecular mechanisms governing this complex disease, offering potential avenues for targeted therapies and diagnostic strategies.

In our current study, we observed a noteworthy increase in the prevalence of mutations within the SNP locus rs2069705 of IFNγ. This finding emerged from the analysis of DNA extracted from 100 labial gland tissues of patients with pSS and 100 samples from healthy individuals. The heightened occurrence of mutations at the rs2069705 locus strongly suggests its potential involvement in the pathogenesis of pSS, further emphasizing the central role of IFNγ in the disease’s etiology. Importantly, mutations at rs2069705 have been associated with elevated levels of IFNγ in the peripheral blood of patients with pSS and greater disease severity in individuals carrying this mutation (32). Moreover, other SNP loci within the IFNγ gene, such as rs1861494, rs2069718, and rs2430561, have shown associations with the risk of developing pSS (33, 34). Collectively, these findings suggest that polymorphisms within the IFNγ gene significantly contribute to pSS pathogenesis. A comprehensive exploration of the intricacies of the IFNγ gene is warranted to shed further light on the foundations of pSS and unveil potential novel diagnostic and therapeutic targets.

Our bioinformatics analysis revealed marked elevations in the expression of both IFNγ and B-cell activating factor (BAFF) in pSS samples compared to normal samples. This observation underscores the critical roles of these factors in pSS pathogenesis. In addition, previous studies have reported significantly elevated levels of IFNγ and BAFF in the saliva and tears of patients with pSS, with strong correlations established between these elevations and disease severity and duration (35). Elevated serum levels of IFNγ and BAFF in patients with pSS have also been documented, with direct correlations observed with disease activity (4). These findings align with our results, further highlighting the significant immunomodulatory roles played by IFNγ and BAFF in pSS pathogenesis.

Furthermore, our study established a significant and positive correlation between the number of CD20+ B cells and the number of cells expressing IFNγ and BAFF proteins within the lacrimal gland tissue of patients with pSS. Notably, heightened numbers of CD20+ B cells and elevated IFNγ levels have been closely associated with the extent of tissue damage (36). This intricate interplay underscores the significance of B cells, IFNγ, and BAFF in pSS pathogenesis, suggesting a complex interrelationship among these elements. A comprehensive investigation into these markers holds promise for advancing our understanding of pSS pathogenesis.

Our study further substantiated the roles of IFNγ and BAFF in the pathogenesis of pSS through in vivo experiments with animal models. We discerned that the suppression of IFNγ significantly reduced BAFF expression and inhibited lymphocyte infiltration in the exocrine gland tissues of pSS mice. Conversely, overexpression of BAFF counteracted the inhibitory effect of IFNγ knockdown. In this context, IFNγ emerges as a key regulator in the pathogenesis of pSS by modulating BAFF expression, revealing a complex interplay between IFNγ and BAFF. Our findings in a mouse model of pSS provide concrete evidence that both IFNγ and BAFF contribute to disease progression, with IFNγ knockdown resulting in reduced disease severity (37). Moreover, the interaction between IFNγ and BAFF proves to be integral to several autoimmune conditions (38). These results align with our observations and collectively reinforce the complex regulatory relationships among IFNγ, BAFF, the JAK/STAT1 signaling pathway, and STAT4 in pSS pathogenesis. This enhanced understanding may open new avenues for the diagnosis and treatment of the disease.

**Figure 5.**
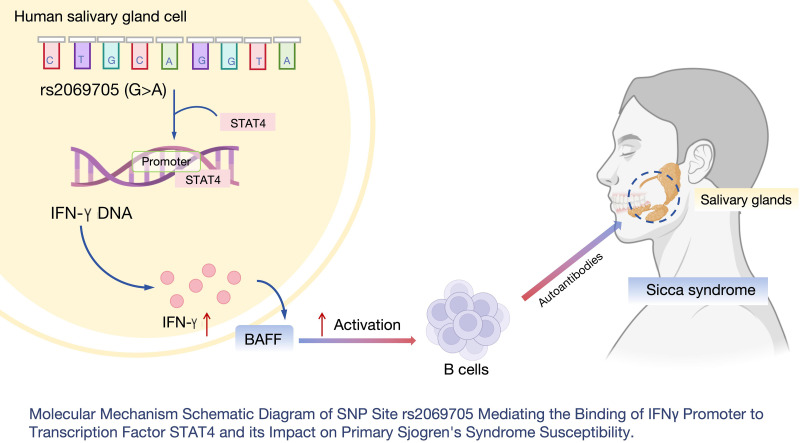
Molecular mechanism of the SNP locus rs2069705 mediating the binding of the IFNγ promoter to the transcription factor STAT4 to affect susceptibility to primary Sjögren’s syndrome. IFNγ, interferon-gamma receptor; SNP, single nucleotide polymorphism; STAT4, signal transducers and activators of transcription 4. Figure created with BioRender.com.

In summary, we tentatively propose that the SNP locus rs2069705 within IFNγ represents a susceptibility site for pSS. This SNP potentially augments IFNγ transcription by facilitating the binding of the IFNγ promoter to the transcription factor STAT4. Consequently, it activates the JAK/STAT1 signaling pathway, leading to increased BAFF expression and fostering B lymphocyte infiltration within lacrimal gland tissues, thereby promoting the development and progression of pSS (Fig. 5). However, our study bears certain limitations. First, our investigation focused solely on a single SNP locus, rs2069705, thus necessitating further exploration of additional susceptibility loci and the potential influence of environmental factors. Second, our examination exclusively encompassed lacrimal gland tissues in pSS mice, thus requiring extension to other exocrine gland tissues and the systemic immune system. Lastly, our study concentrated on only two key factors, IFNγ and BAFF, prompting the need for additional scrutiny of other signaling pathway molecules.

## ETHICAL APPROVALS

The study was conducted following the relevant guidelines and approved by the animal ethics committee of Mianyang Central Hospital.

## DATA AVAILABILITY

The data that supports the findings of this study are available on request from the corresponding author.

## DISCLOSURES

No conflicts of interest, financial or otherwise, are declared by the authors.

## AUTHOR CONTRIBUTIONS

H.L. and Y.J. performed experiments; M.L. and J.S. analyzed data; J.S. interpreted results of experiments; H.L. and J.S. prepared figures; X.C. and M.L. edited and revised manuscript; X.C. and Y.J. approved final version of manuscript.
